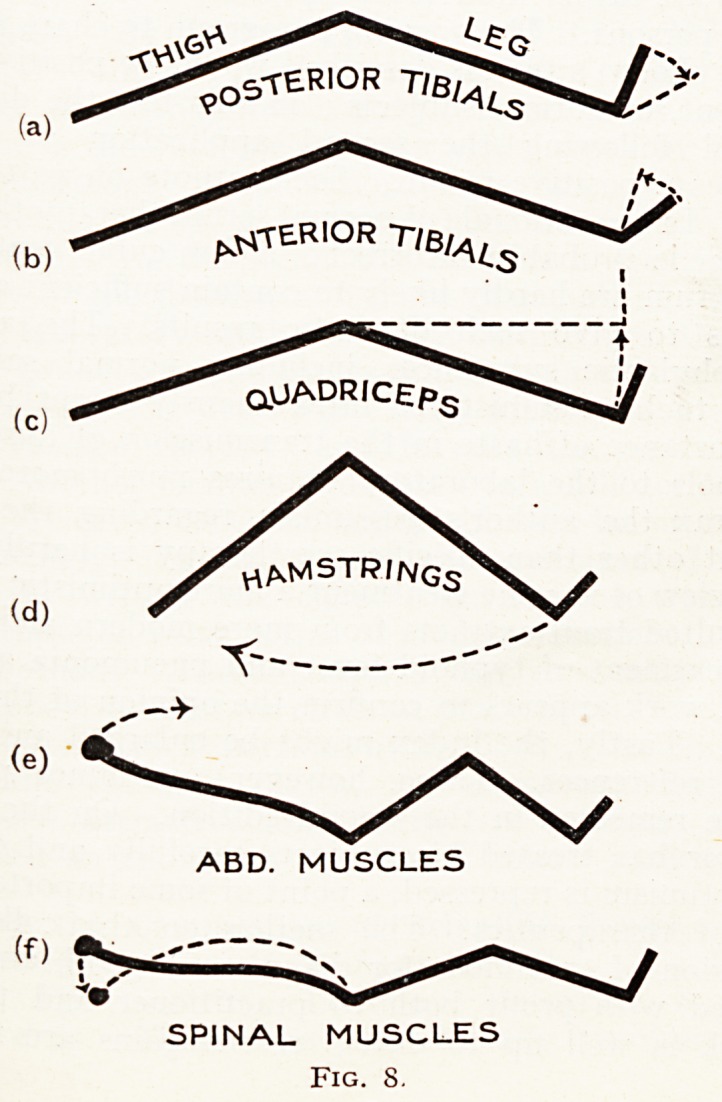# A Few Suggestions in General Surgery
1Read before a Meeting of the Bristol Medico-Chirurgical Society on May 14th, 1924.


**Published:** 1924-10

**Authors:** C. F. Walters

**Affiliations:** Surgeon to the Bristol Royal Infirmary


### A FEW SUGGESTIONS IN GENERAL SURGERY.[1]

BY

C. F. Walters, F.R.C.S.,

*Surgeon to the Bristol Royal Infirmary.*

---

### Pressure Forceps Technique.

My first suggestion, one on general technique, proven this seven years, is based on a single page article of some ten years ago that I have been unable to unearth again, in which is described a method of tying ligatures by means of artery forceps. On this one has developed a technique by which all manœuvres of ligaturing and suturing are done by means of pressure forceps, so that one may perhaps describe it as "The Pressure Forceps Technique."

One has always seen in the so-called "spoon and fork" operation on bone, brain and joint an element of risk introduced by hand ligaturing, and sometimes one has seen the operation still further jeopardised by hand suturing when gloves are so readily torn, whereas by means of the Pressure Forceps Technique the surgeon's hands approach no nearer to the wound at any period of the operation than the distal end of the instrument in use. This applies also to the house surgeon and dressers. So that as far as the surgeon is concerned, from an aseptic point of view, the chain is complete, and bone, brain and joint operations can be embarked on with complete confidence.

This is the most important feature of this method, but there are others.

*Accessibility* is improved; it is easy to ligature at a depth by sight when hand ligature must of necessity obscure the view, and is sometimes impossible.

[1] Read before a Meeting of the Bristol Medico-Chirurgical Society on May 14th, 1924.

*While this method is best shown by cinema, with the written word one is confined to the following diagrams:—*

FIG. 1.

*When second movement of right hand forceps is complete it has seized right end of ligature, it then passes to the right and the left to the left.*

FIG. 2.

*After this movement of the right-hand forceps is complete, and it has seized right end of ligature, the left-hand forceps passes to the right and the right-hand forceps to the left to complete the first loop.*

*Facility* is greater; one can readily tie a ligature with one and a half inches at one end and a quarter of an inch at the other.

*Rapidity* is increased, forceps ligature being at least half as fast again as by hand.

*Economy* is a marked feature; it is possible to tie seven or eight times as many ligatures with the same length of catgut by forceps as by hand, and when the yearly hospital bills for catgut are so large it is worth considering.

The complete method is applicable to all surface operations and the tying and suturing to many deep ones.

Lastly, the mental comfort of having thus dealt with bone, brain and joint, in fact any operation where a perfect aseptic technique is obtained, is alone in my opinion well worth the while.

### Abdominal Drainage.

Next I would suggest that a dental rubber dam drain down to but not through the peritoneum and left in position for six or seven days is, *so to speak*, an antidote to incisional herniæ in septic appendix wounds in particular, and septic abdominal wounds generally, by which I mean wounds inevitably soiled owing to the presence of free pus in the peritoneum.

My clinical impression—and one knows what such are worth—after utilising this method for the last three years was that suppuration was prevented or diminished, a sounder wound and healing by first intention more frequently obtained where free pus was present in the abdomen.

In an attempt to prove this I took two periods, the past three years, during which I have used a dental rubber dam drain, and the three years previous to that, during which no drain was introduced into the appendix wound itself.

In some 300 cases of appendicitis on which I had operated during both periods 41 cases during the first where no drain was used were recorded as having free pus in the abdomen, and 54 in the second period, when rubber dam was utilised.

Despite many personal visits to their houses in an attempt to see the wounds, I was able to see only 25 out of the 95 in which the abdomen contained free pus at the time of operation.

Of these, 8 were in the first period without drain and 17 in the second period with it. Of the 8 seen without drain four had well-marked herniæ, of the 17 with drain only one had a hernia. These figures are far too small to prove anything, but I would suggest that where a wound is necessarily infected by the presence of free pus in the abdomen a dental rubber dam drain down to but not through the peritoneum and left *in situ* for six or seven days will in such cases tend to lessen the incidence of incisional hernia.

Of my next two suggestions one carries no proof for lack of material and the other needs more experience, but I venture to put them forward in the hope that if they are worthy of consideration others may test their worth or lack of it.

### Exteriorisation.

First, as to the thorax. Those who have delved much in thoracic surgery, and for my own part I have tried most known and some unknown methods, will agree as to the difficulty in closing chronic empyema cavities, whether it be by Estlander, Schede, decortication, discission, sub-costal pleural freeing, and what not.

The difficulties of pre-operative sterilisation, the failure of lung to remain distended that expanded freely at operation, recurrent suppuration months after an apparently successful healed decortication—sometimes the complete failure of the procedure—and on one occasion, in my own experience, a fatal ending, may bring one's efforts to naught.

For the larger cavities I have nothing new to offer; but for the smaller, those of fist or sausage size and shape which sometimes fail to close despite apparently adequate drainage, I would suggest what I have called *Exteriorisation*, though my experience is based on one case only—that of a young man with a cavity of some months' standing due to collapse of the left lower lobe following empyema.

For weeks I attempted to sterilise this as a preliminary to decortication, and failed, and in despair turned up the whole anterior wall of the cavity, sub-periosteally excising sections of three ribs, sewing the flap to the chest wall above, and packing with Eusol the completely-exposed cavity. To my astonishment it rapidly granulated, the flap tore out its fixing stitches, replaced itself and healed.

Eighteen months later I saw him; he was doing his usual work with ease, and although the upper lobe only was functioning the wound had remained soundly healed.

### The Dural Flap.

Not infrequently one is asked by one's medical colleagues to do a decompression for symptoms of cerebral tumour, and one has not failed to note the anxiety of the physician that the surgeon should stop at the dura, fearing—not unjustly—cerebral hernia if it be opened.

Sometimes one has respected this wish and sometimes disregarded it with regret, but if the dura can be opened and readily closed without fear of hernia it may relieve the physician of his fears and justify the curiosity of the surgeon.

One is aware that cerebral tension can be diminished by ventricular puncture, though this is not without danger and may fail, and one has found that hypertonic saline will deplete the brain of fluid; but I would suggest what may be described as the *Dural flap* as a means by which cerebral hernia can be prevented, the dura readily restored with more

FIG. 3.
*Superficial Flap Continuous Line—Deep Dotted.*

FIG. 4.
*Superficial Flap Raised.*

FIG. 5.

*Both Flaps-of-Dura Everted.*

FIG. 6.
*Flaps Closed.*

ample decompression, the physician comforted and the surgeon almost satisfied.

### Method of Flap Cutting.

The dura having been exposed with a very sharp broad-bellied scalpel, the flat surface of which is held almost parallel to the dura approaching it at a very slight angle, the outer surface of the membrane is raised following the outline of the flap (Fig. 3) for about a sixteenth of an inch. This flap is then seized with finely-toothed forceps and pulled on, when the dura will be found to split readily into two layers attached to one another by fine fibrous processes.

If the knife is held as before mentioned and drawn across these fine processes the splitting can be fairly rapidly proceeded with, and sometimes further facilitated by stripping with gauze held in forceps.

The first flap having been raised (Fig. 4), of such a shape as the diagram shows, the deeper layer of the dura is then divided as indicated (Fig. 5), when sufficient redundant membrane will be found not only to readily cover the protruding brain tissue, but to allow also an increased space for decompression (Fig. 6).

Since this method occurred to me some three months ago I have had four opportunities of testing it, two on children and two on adults.

I may say at once it fails in the child, its dura is too thin for splitting, but fortunately is more elastic and more readily closed than that of the adult. In the adults it proved easy and was successful.

One adult died a month after operation, and the portion of dura in which the flap had been cut was obtained and the finding somewhat unexpected. The under surface of the superficial flap, that with a raw surface, was perfectly smooth, while that of the lower flap, the true inner dural surface, was slightly rough, but nowhere was either flap adherent to the brain.

RECUMBENT EXERCISES DURING CONVALESCENCE.

Lastly, as to convalescence—and this should apply not only to surgical but to medical cases also. It becomes the practice in surgery more and more to lessen the time, following operation, during which a patient is what one may call bed-ridden.

Frequently the simple appendix patient is on his or her feet at the end of a week ; some surgeons get them up still earlier, and some would, if they could, walk them off the operation table ; but whether it be one, two, or three weeks that a patient must be kept in bed muscular power and tone is diminished from disuse, and after longer periods the first progress from bed to chair is only too often a tottering effort needing much external support.

This I suggest is unnecessary and can be largely eliminated by practising, as I have for some period, what I have called *Recumbent Exercises*, by which when the patient is allowed to get up he can literally, though unable to manage the middle of the quotation, arise and walk.

The most gentle manner in which these exercises can be applied to the aged and debilitated is in the form of deep breathing, five deep breaths after each meal, increasing by one or more daily at each period, and so improving oxygenation and tending to prevent hypostatic congestion and possibly other complications. The exercises in their complete form bring into play every important body muscle, and in the increasing doses mentioned not only maintain muscular tone but actually in the fat and indolent better their pre-operative muscular condition and, moreover, give them something to think about.

The whole series can be done in the supine position, and although suitable exercises will at once occur to any medical man, I can illustrate those that I use by the following rough diagrams (Fig. 8).

FIG. 8.

## Figures and Tables

**Fig. 1. f1:**
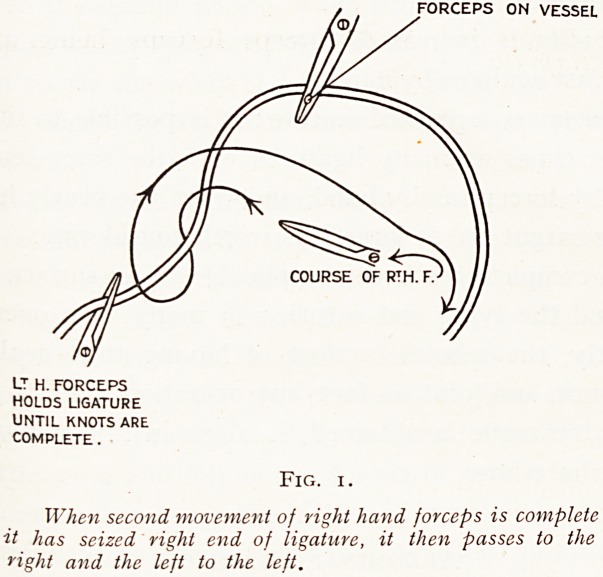


**Fig. 2. f2:**
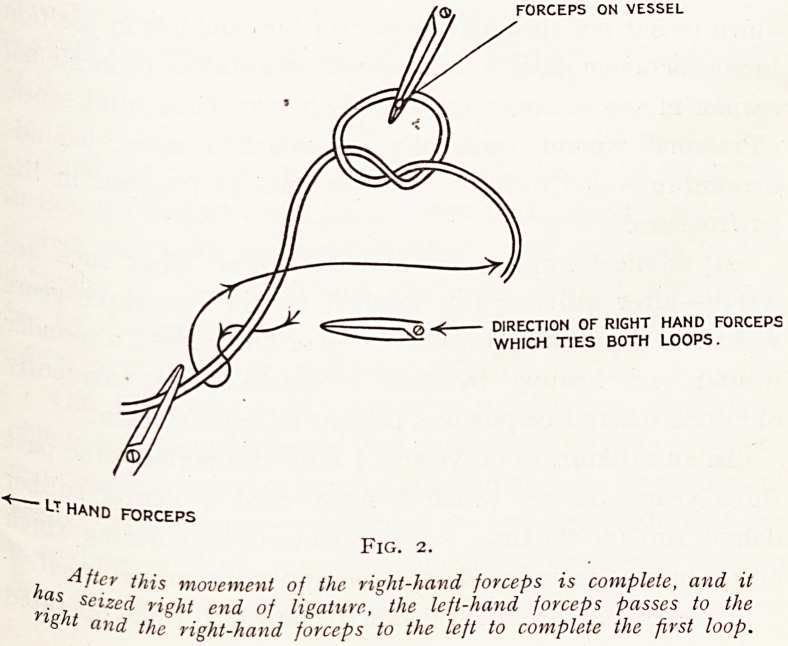


**Fig. 3. f3:**
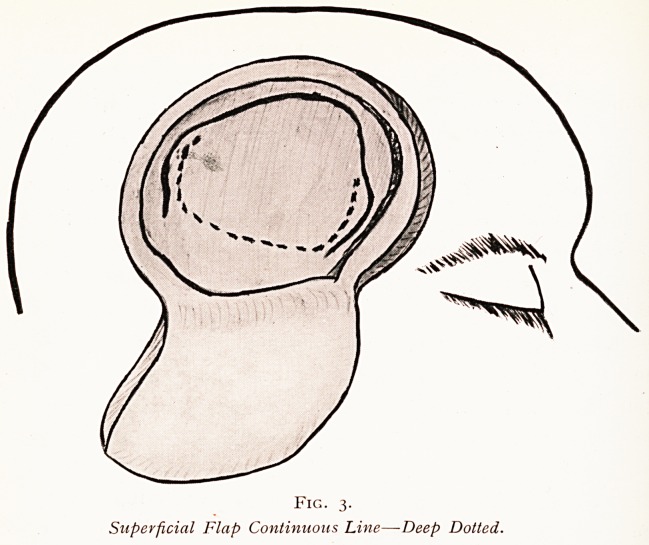


**Fig. 4. f4:**
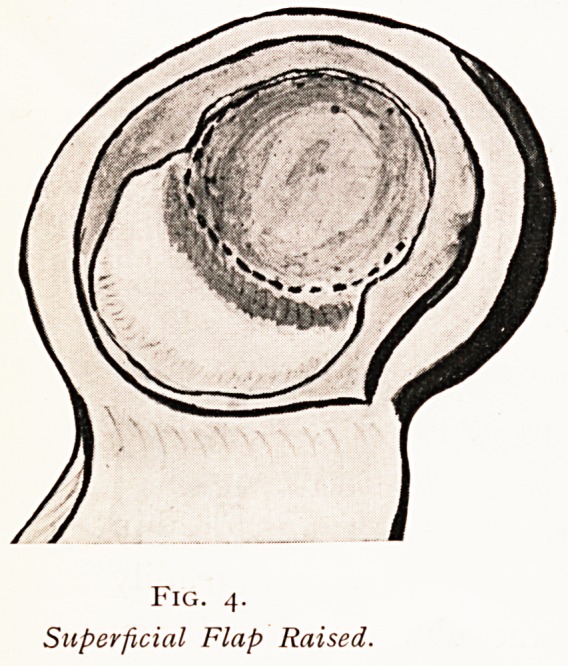


**Fig. 5. f5:**
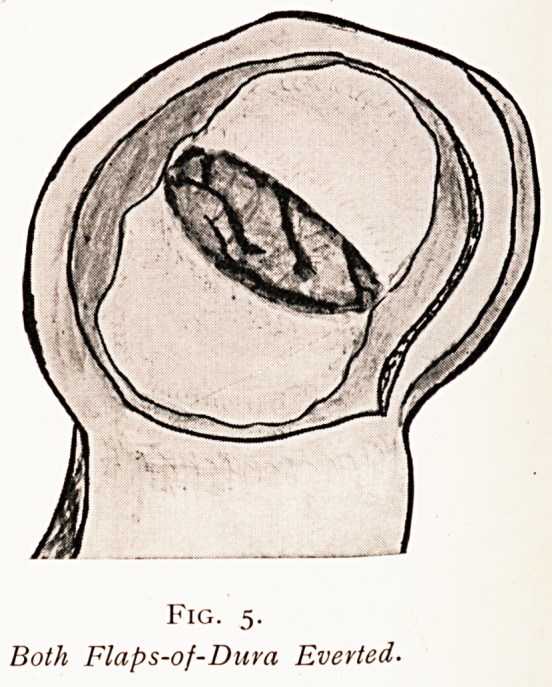


**Fig. 6. f6:**
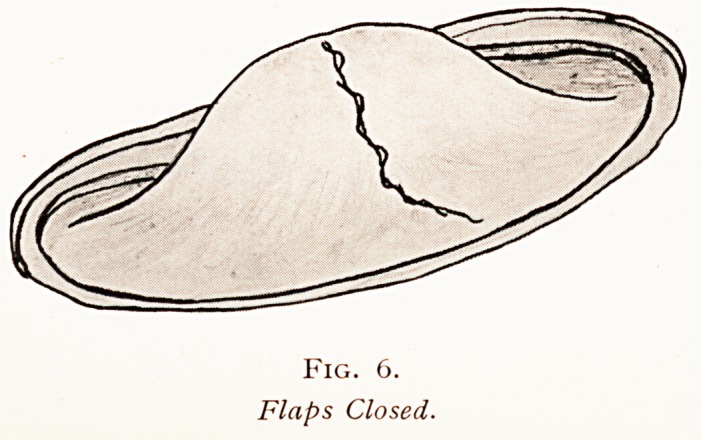


**Figure f7:**
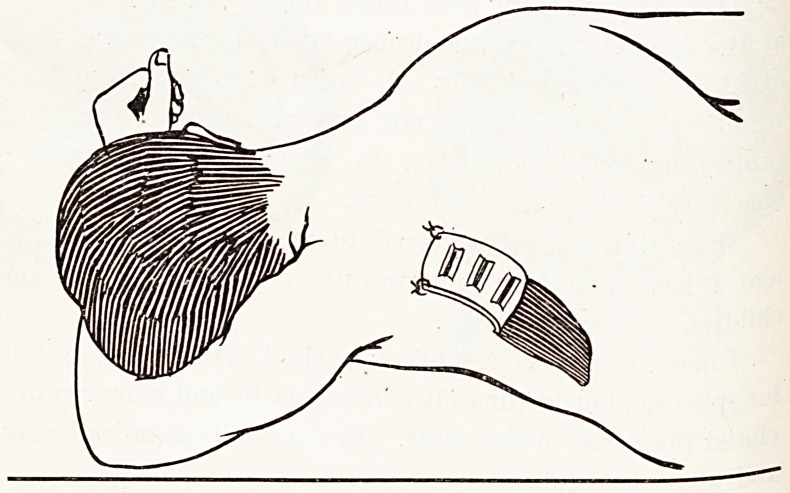


**Fig. 8. f8:**